# High‐throughput monoclonal gammopathy community monitoring programme

**DOI:** 10.1111/bjh.70366

**Published:** 2026-02-12

**Authors:** Gaurav Agarwal, Lauren Campbell, Oluremi Carty, Jemma Larham, Elizabeth Knight, Sally Moore, Sarah Gooding, Jaimal Kothari, Joe Browning, Julia Evans, Lisa Ferguson, Ana Vieira, Liam Swanborough, Pamela Roberts, Elizabeth Bateman, Ross Sadler, Karthik Ramasamy

**Affiliations:** ^1^ Division of Hematology/Oncology, Boston Children's Hospital Harvard Medical School Boston Massachusetts USA; ^2^ Oxford Translational Myeloma Centre, Nuffield Department of Orthopaedics, Rheumatology and Musculoskeletal Sciences (NDORMS) University of Oxford Oxford UK; ^3^ Immunology Laboratory Oxford University Hospitals NHS Foundation Trust Oxford UK; ^4^ Department of Haematology Oxford University Hospitals NHS Foundation Trust Oxford UK; ^5^ Medical Sciences Division University of Oxford Oxford UK; ^6^ Department of Haematology University Hospitals Bristol and Weston NHS Foundation Trust Bristol UK; ^7^ MRC Molecular Haematology Unit, Weatherall Institute of Molecular Medicine University of Oxford Oxford UK

**Keywords:** mgus, monitoring, myeloma, surveillance, system

To the Editor,

Monoclonal gammopathy (MG) is present in 4.5% of adults older than 50 years.^1^ Given an annual 1% rate of progression to multiple myeloma (MM),^2^ regular MG monitoring has the potential to improve MM outcomes through earlier diagnosis.^3^ To this end, a variety of surveillance systems have been implemented in the National Health Service (NHS), including hospital, primary care and nurse‐led surveillance.^4^ However, as the vast majority of new MG patients are low risk and unlikely to progress, most services are inefficient and unsustainable to support population‐based monitoring.^5^ There is a need for a robust high‐throughput system that can monitor most patients in the community, while streamlining high‐risk MG for review in secondary care.

Here, we describe a risk‐adapted MG community monitoring programme (OxCOM). Patients with new incidental MG (iMG, in contrast to screened MG) in Thames Valley were stratified and prospectively monitored. Patients with a new iMG were captured over a 24‐month period (between 4 March 2021 and 3 March 2023) in an Immunology Laboratory covering a population of 2.5 million people in Oxfordshire. During its conception and design, OxCOM was discussed with key stakeholders, including general practitioners (GPs), haematology registrars and a MM/MG patient focus group.

Under OxCOM, iMG cases were risk stratified into low‐, intermediate‐ or high‐risk groups. Triaging followed expert consensus criteria (Figure 1) and was achieved using a combination of automated algorithms on a task management software, together with daily review by the attending senior scientist in the central laboratory. All senior scientists received structured training to ensure consistent and safe implementation. In addition to existing infrastructure, implementation of OxCOM required only one additional laboratory staff member, primarily for administrative tasks such as issuing patient letters and completing background safety checks.

**FIGURE 1:**
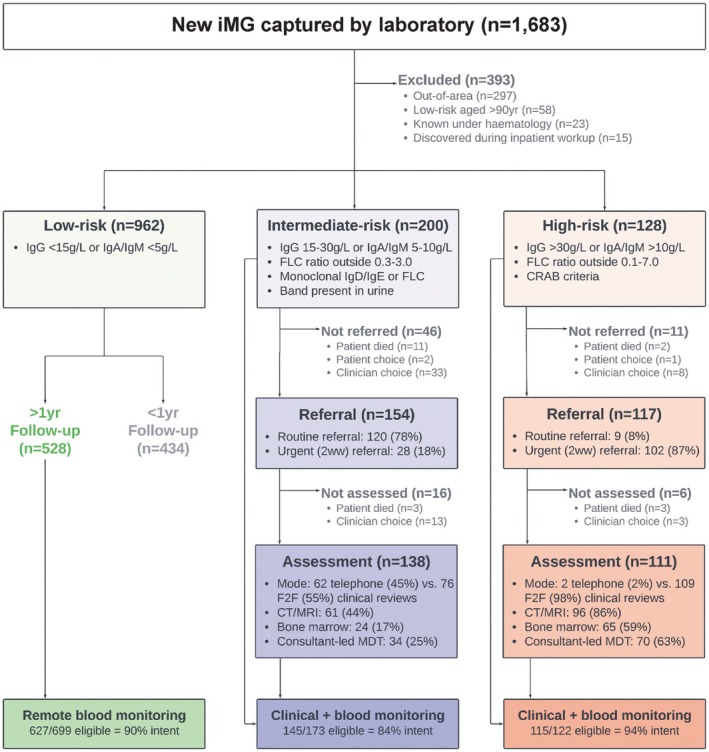
Consort diagram of iMG detection, referral and assessment. OxCOM risk stratification strategy for incidental monoclonal gammopathy (iMG), and patient flow during 2 years of follow‐up. 2ww, two‐week‐wait urgent suspected cancer referral; CRAB, hypercalcaemia, renal impairment, anemia or bone lesions; CT, computed tomography; F2F, face‐to‐face; FLC, serum free light chains; MDT, multidisciplinary team; MRI, magnetic resonance imaging; *n*, number of patients.

Following stratification, the central laboratory recommended risk‐adapted follow‐up. Patients were excluded from analysis if out of area, aged 90 years or older with low‐risk iMG, under regular haematology review or had malignancy diagnosed as an inpatient following iMG detection. Low‐risk iMG patients were advised to undergo regular blood monitoring by primary care every 4–12 months, without clinical review (OxCOM Remote). Intermediate patients were advised for regular blood monitoring and clinical review by secondary care, in a virtual telephone clinic where possible (OxCOM Telemed). High‐risk patients were recommended for urgent face‐to‐face assessment by haematology services (OxCOM F2F).

Results from primary care iMG testing were communicated to the GP via standard reporting systems, accompanied by guidance on recommended next step. For iMG detected through secondary care testing, results were sent to the requesting clinician as well as the patient's GP. The requesting clinician informed the patient; after 4 weeks, a welcome letter was automatically sent to the patient, which outlined the planned monitoring schedule. To facilitate clinician and patient engagement, OxCOM was publicised through written materials, email communications and a dedicated website.

One thousand two hundred and ninety patients had iMG detected over a 24‐month period [962 low risk (75%), 200 intermediate risk (16%) and 128 high risk (9%)] (Figure 1). 50% were female and the median age was 75.4 years (Table S1). Testing was requested equally between primary and secondary care (50% each); the most common indications were anaemia (13%), bone pain (13%) and fracture (13%), all of which were over‐represented in intermediate‐ and high‐risk iMG [*p* = 0.0002]. Of individuals with high‐risk iMG, 91% were referred for review by the haematology service, the majority of which were requested as an urgent review within 2 weeks (87%). Of intermediate‐risk patients, 77% were referred, with the majority of requests for routine review (78%).

We assessed the patterns of iMG clinical assessment. The majority of high‐risk patients were reviewed face to face (98%) and by a Consultant Haematologist (68%), while a greater proportion of intermediate‐risk patients were assessed in a virtual telephone clinic (45%) that was primarily nurse and physician associate‐led [*p* < 0.0001]. Following clinical assessment, a greater percentage of high‐risk than intermediate‐risk patients received investigations, including CT/MRI (86% vs. 44%), bone marrow biopsy (59% vs. 17%) and MDT discussion (63% vs. 25%) [*p* < 0.0001]. Therefore, OxCOM achieves a high degree of patient follow‐up in secondary care for intermediate‐ and high‐risk patients and distributes healthcare resource towards higher risk iMG patients.

Next, we evaluated the fidelity of repeat monitoring. We assessed intent for monitoring as the proportion of patients receiving subsequent surveillance within 4 months (IgA or IgM) or 12 months (IgG), with a 2‐month grace period. Follow‐up rates were compared to a reference cohort of new iMG detected in 2019 (prior to set‐up of OxCOM, in which monitoring was managed in the community) that were risk stratified retrospectively. Under OxCOM, of 525 low‐risk patients with ≥12‐month follow‐up, 475 (90%) had received repeat monitoring within an appropriate time interval (compared to 37% in 2019) (Figure 2A). Similarly, 84% of intermediate‐risk and 94% of high‐risk patients received further follow‐up, compared to 77% and 75% in 2019 respectively. There was also clinician‐led reclassification of patient risk (Figure 2B). Of 253 patients initially assessed by secondary care, 65 (26%) had been transferred to remote primary care monitoring after a median of three clinical reviews and 7.3 months of follow‐up. Therefore, OxCOM enables a significantly increased fidelity of repeat intent for monitoring in the low‐risk iMG patients and enables dynamic risk designation informed by ongoing clinical and biochemical evaluation.

**FIGURE 2:**
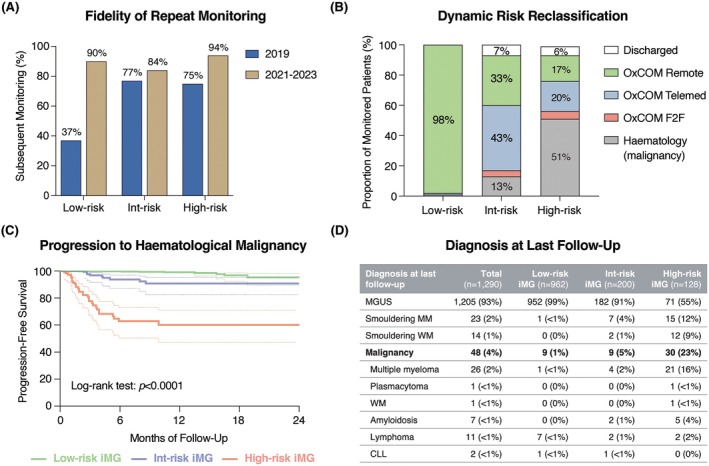
OxCOM achieves high fidelity of follow‐up with appropriate risk stratification. (A) Fidelity of repeat intent for monitoring of incidental monoclonal gammopathy (iMG)—defined by repeat paraprotein measurement or clinical assessment of iMG—comparing between 2019 (pre‐OxCOM) and 2021–2023 (post‐OxCOM), by risk group. (B) Last known follow‐up status of individuals initially triaged as low‐, intermediate‐ or high‐risk iMG, showing dynamic risk reclassification by clinician judgement. F2F = face to face. (C) Kaplan–Meier graph showing progression to haematological malignancy in individuals initially triaged as low‐, intermediate‐ or high‐risk iMG. Log‐rank test. (D) Diagnosis at last follow‐up for individuals under OxCOM, by initial risk stratification. CLL, chronic lymphocytic leukaemia; MM, multiple myeloma; WM, Waldenström macroglobulinaemia.

We next assessed the diagnostic yield of iMG assessment and longitudinal follow‐up. At a median follow‐up of 12.2 months, 37 patients (3%) were diagnosed with smouldering myeloma or Waldenström macroglobulinaemia (WM) and remained under observation, while 48 of 1290 individuals were diagnosed with a haematological malignancy (corresponding to 1% of low‐risk, 5% of intermediate‐risk and 23% of high‐risk iMG cases) (Figure 2C,D). These diagnoses included 34 patients (3%) with multiple myeloma, amyloidosis or Waldenström macroglobulinaemia (WM) requiring treatment and 11 patients (1%) with lymphoma. Importantly, 46 of 48 (96%) haematological malignancies were diagnosed through outpatient review and investigation, with only two cases (4%) identified during an inpatient admission following a new disease‐related presentation after initial iMG detection. These findings indicate that the vast majority of malignancies were diagnosed in a setting of relative clinical stability, arguing against clinically significant diagnostic delays attributable to OxCOM‐initiated follow‐up and supporting the safety of this monitoring strategy.

We next examined whether malignancy diagnoses arose directly from OxCOM‐initiated monitoring or were made independently of this framework. When stratified by cancer type (Figure S1A), most multiple myeloma diagnoses were detected through OxCOM monitoring (22/26), whereas the majority of lymphoma cases were diagnosed outside OxCOM, typically following lesion detection on imaging performed in secondary care (7/11). Consistent with this, all malignancies arising in low‐risk iMG patients were diagnosed independently of OxCOM, reflecting the enrichment of lymphoma (7/9) within this group (Figure S1B). In contrast, 25 of 30 (83%) high‐risk iMG patients who progressed to malignancy were diagnosed through OxCOM‐initiated review, consistent with both the predominance of multiple myeloma in this group (21/30) and expedited clinical assessment triggered by biochemical or clinical criteria. Among intermediate‐risk iMG patients, approximately half of malignancies were detected through OxCOM monitoring (5/9). Taken together, these data demonstrate that OxCOM‐initiated monitoring contributes substantially to the diagnosis of plasma cell dyscrasias, in which paraprotein dynamics closely track disease activity. Conversely, in lymphoid malignancies such as lymphoma and chronic lymphocytic leukaemia (CLL), where the paraprotein may represent a more passive biomarker, diagnoses are more commonly made through conventional clinical pathways.

Population‐based screening estimates suggest that around 1.3 million adults in the United Kingdom may have iMG.^1^ While universal iMG screening may have potential value, the risk of progression varies significantly, and a high‐throughput system is required to triage individuals and enable high fidelity of subsequent follow‐up. Traditionally, the burden of iMG monitoring has fallen on primary care^6^ and has suffered from high loss to follow up. OxCOM is a supported service for primary care, in which initial stratification and triaging are done in a central laboratory hosted by secondary care, and the intensity of follow‐up is matched to the risk of progression. Our 2‐year experience of a risk‐adapted iMG monitoring system has enabled appropriate allocation of healthcare resources towards higher risk patients. Moreover, OxCOM maintained high fidelity of repeated follow‐up for individuals with low‐risk iMG, which were relatively enriched for the diagnosis of subsequent lymphoma. Intermediate‐risk patients—which may not traditionally meet criteria for urgent haematology referral—were able to benefit from structured clinical review, typically as a virtual telephone appointment, and could be assessed for further investigations or face‐to‐face review or reassured and moved into remote blood monitoring. Therefore, OxCOM enables a risk‐stratified system for high‐throughput monitoring of iMG in the community.

There are some limitations in our analysis. First, as a relatively new service, there are only 2 years of follow‐up data available; much remains to be understood, such as the natural history of iMG between subpopulations and the incremental diagnostic yield of OxCOM follow‐up particularly in low‐ and intermediate‐risk iMG cases. High throughput of patients will enable the service to be refined iteratively, for example, through adapting risk thresholds and monitoring frequencies. Second, while OxCOM criteria stipulate a repeat screening 4 months after discovery (to capture dynamic risk in patients with a paraprotein on an upward trajectory), we have not performed this in those with IgG isotype iMG, as OxCOM has rigorously stratified low‐risk iMG at detection. Third, as intent to monitor analysis was compared to a retrospective dataset in 2019, this comparison may be subject to confounding factors; nevertheless, as service demands have increased since the COVID‐19 pandemic, the improved fidelity of follow‐up of OxCOM in spite of this argues in favour of its efficiency.

In summary, we demonstrate the feasibility of a risk‐adapted high‐throughput clinical monitoring service for iMG. Our approach has high fidelity, low cost and a relevant patient population in a safe and efficient way. The service, therefore, establishes an iterative model to enable tailored follow‐up and monitoring of iMG, which could be adopted for sustainable surveillance of precursor states more generally.

## AUTHOR CONTRIBUTIONS

**Gaurav Agarwal:** Conceptualization; methodology; data curation; formal analysis; investigation; writing – original draft; writing – review and editing. **Elizabeth Knight:** Investigation; data curation. **Jemma Larham:** Investigation; data curation. **Ross Sadler:** Supervision; conceptualization; methodology; writing – review and editing. **Karthik Ramasamy:** Supervision; conceptualization; methodology; writing – review and editing. All remaining coauthors were involved in routine data collection as part of the running of OxCOM. All authors read and approved the final version of the manuscript.

## Supporting information


Figure S1.

Table S1.

## Data Availability

The data that support the findings of this study are available on request from the corresponding author. The data are not publicly available due to privacy or ethical restrictions.
