# Supplementary figures and images for: High‐throughput monoclonal gammopathy community monitoring programme
Source: Br J Haematol. 2026 Feb 12;208(5):1826–30. doi: 10.1111/bjh.70366 (PMC13176512; doi:10.1111/bjh.70366)

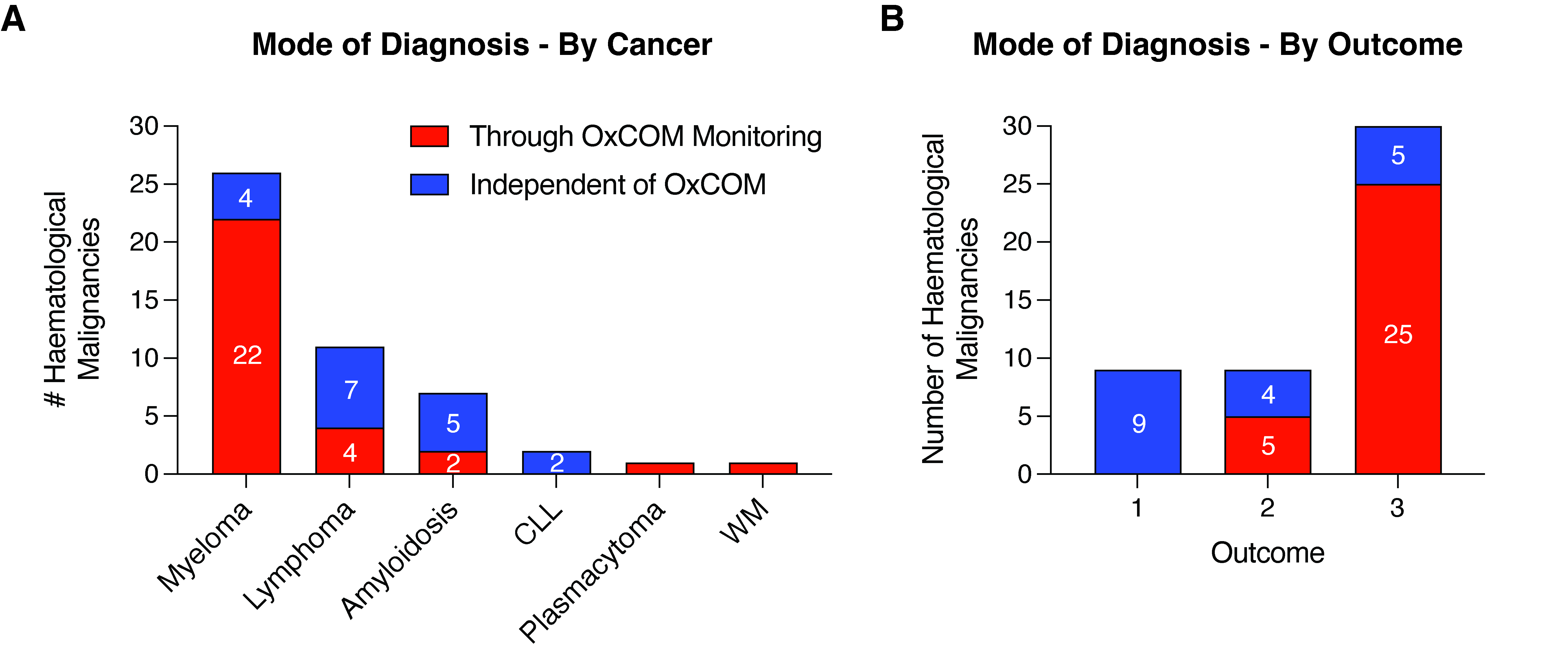

Supplement: Supplementary file 1 — Figure S1. [file BJH-208-1826-s001.tif]
