# Supplementary material for: High‐throughput monoclonal gammopathy community monitoring programme
Source: Br J Haematol. 2026 Feb 12;208(5):1826–30. doi: 10.1111/bjh.70366 (PMC13176512; doi:10.1111/bjh.70366)
Supplement: Supplementary file 2 — Table S1. [file BJH-208-1826-s002.docx]

**Supplementary Table 1**

|  | Total | Low-risk | Int-risk | High-risk | *P* |
| --- | --- | --- | --- | --- | --- |
| Capture (n) | **1,290** | **962** | **200** | **128** |  |
| Age (years), median [SD] | 75.4 [12.8] | 74.6 [12.7] | 78.6 [12.6] | 76.6 [12.8] | <0.0001 |
| Female sex (%) | 645 (50%) | 477 (50%) | 98 (49%) | 70 (55%) | 0.5295 |
| Requesting clinician |  |  |  |  |  |
| Primary care | 553 (43%) | 397 (41%) | 94 (47%) | 62 (48%) | 0.0958 |
| Secondary care | 552 (43%) | 397 (41%) | 92 (46%) | 63 (49%) |  |
| Unknown | 185 (14%) | 168 (17%) | 14 (7%) | 3 (2%) |  |
| Reason for test |  |  |  |  |  |
| Anemia | 162 (13%) | 91 (9%) | 43 (22%) | 28 (22%) | 0.0002 |
| Bone / back pain | 166 (13%) | 100 (10%) | 32 (16%) | 34 (27%) |  |
| Constitutional symptoms | 148 (11%) | 109 (11%) | 26 (13%) | 13 (10%) |  |
| Fracture | 173 (13%) | 116 (12%) | 36 (18%) | 21 (16%) |  |
| Incidental (liver/biologics) | 82 (6%) | 70 (7%) | 7 (4%) | 5 (4%) |  |
| Renal impairment | 43 (3%) | 28 (3%) | 9 (5%) | 6 (5%) |  |
| Other | 305 (24%) | 220 (23%) | 54 (27%) | 31 (24%) |  |
| Unknown | 300 (23%) | 268 (28%) | 27 (14%) | 5 (4%) |  |
| Paraprotein isotype |  |  |  |  |  |
| IgG | 825 (64%) | 709 (74%) | 71 (36%) | 45 (35%) | <0.0001 |
| IgA | 124 (10%) | 61 (8%) | 39 (20%) | 24 (19%) |  |
| IgM | 219 (17%) | 152 (16%) | 42 (21%) | 25 (20%) |  |
| IgD | 2 (<1%) | 0 (0%) | 0 (0%) | 2 (2%) |  |
| Free light chain | 78 (6%) | 7 (1%) | 39 (20%) | 32 (25%) |  |
| Multiple clones | 42 (3%) | 33 (3%) | 9 (5%) | 0 (0%) |  |
| Referral (n) | **323 (25%)** | **52 (5%)** | **154 (77%)** | **117 (91%)** |  |
| Routine | 161 (50%) | 32 (62%) | 120 (78%) | 9 (8%) | <0.0001 |
| Urgent (2ww) | 143 (44%) | 13 (25%) | 28 (18%) | 102 (87%) |  |
| Unknown | 19 (6%) | 7 (13%) | 6 (4%) | 6 (5%) |  |
| Assessment (n) | **283 (22%)** | **34 (4%)** | **138 (69%)** | **111 (87%)** |  |
| Telephone | 71 (25%) | 7 (21%) | 62 (45%) | 2 (2%) | <0.0001 |
| F2F | 212 (75%) | 27 (79%) | 76 (55%) | 109 (98%) |  |
| Assessing clinician |  |  |  |  |  |
| CNS / PA | 78 (28%) | 3 (9%) | 68 (49%) | 7 (6%) | <0.0001 |
| Registrar | 80 (28%) | 15 (44%) | 36 (26%) | 29 (26%) |  |
| Consultant | 125 (44%) | 16 (47%) | 34 (25%) | 75 (68%) |  |
| Investigations |  |  |  |  |  |
| CT / MRI | 178 (63%) | 21 (62%) | 61 (44%) | 96 (86%) | <0.0001 |
| Bone marrow | 101 (36%) | 12 (35%) | 24 (17%) | 65 (59%) | <0.0001 |
| Consultant-led MDT | 117 (41%) | 13 (38%) | 34 (25%) | 70 (63%) | <0.0001 |
